# Childhood growth outcomes 2 years after hypertensive versus normotensive pregnancy: a P4 study

**DOI:** 10.1038/s41390-023-02789-7

**Published:** 2023-09-06

**Authors:** Megan L. Gow, Priya Vakil, Lynne Roberts, Greg Davis, Joseph M. Khouri, Ana Dosen, Mark A. Brown, Maria E. Craig, Amanda Henry

**Affiliations:** 1 Discipline of Paediatrics and Child Health, School of Clinical Medicine, UNSW Medicine and Health, Sydney, NSW Australia; 2 Department of Women’s and Children’s Health, St George Hospital, Sydney, NSW Australia; 3 The University of Sydney Children’s Hospital Westmead Clinical School, Sydney, NSW Australia; 4 Discipline of Women’s Health, School of Clinical Medicine, UNSW Medicine and Health, Sydney, NSW Australia; 5 St George and Sutherland Clinical Campus, School of Clinical Medicine, UNSW Medicine and Health, Sydney, NSW Australia; 6 Renal Medicine, St George Hospital, Sydney, NSW Australia; 7 Department of Paediatrics, St George Hospital, Sydney, NSW Australia; 8 The George Institute for Global Health, Sydney, NSW Australia

## Abstract

**Background:**

Intrauterine exposure to hypertensive disorders of pregnancy, including gestational hypertension (GH) and preeclampsia (PE), may influence infant growth and have long-term health implications. This study aimed to compare growth outcomes of infants exposed to a normotensive pregnancy (NTP), GH, or PE from birth to 2 years.

**Methods:**

Infants were children of women enroled in the prospective Postpartum Physiology, Psychology and Paediatric (P4) cohort study who had NTP, GH or PE. Birth, 6-month (age-corrected) and 2-year (age-corrected) weight z-scores, change in weight z-scores, rapid weight gain (≥0.67 increase in weight z-score) and conditional weight gain z-scores were calculated to assess infant growth (NTP = 240, GH = 19, PE = 66).

**Results:**

Infants exposed to PE compared to NTP or GH had significantly lower birth weight and length z-scores, but there were no differences in growth outcomes at 6 months or 2 years. GH and PE-exposed infants had significantly greater weight z-score gain [95% CI] (PE = 0.93 [0.66–1.18], GH = 1.03 [0.37–1.68], NTP = 0.45 [0.31–0.58], *p* < 0.01) and rapid weight gain (GH = 63%, PE = 59%, NTP = 42%, *p* = 0.02) from birth to 2 years, which remained significant for PE-exposed infants after confounder adjustment.

**Conclusion:**

In this cohort, GH and PE were associated with accelerated infant weight gain that may increase future cardiometabolic disease risk.

**Impact:**

- Preeclampsia exposed infants were smaller at birth, compared with normotensive pregnancy and gestational hypertension exposed infants, but caught up in growth by 2 years of age.
- Both preeclampsia and gestational hypertension exposed infants had significantly accelerated weight gain from birth to 2 years, which remained significant for preeclampsia exposed infants after adjustment for confounders including small for gestational age.
- Monitoring of growth patterns in infants born following exposure to a hypertensive disorder of pregnancy may be indicated to prevent accelerated weight gain trajectories and obesity.

## Introduction

Approximately 5–10% of pregnancies worldwide are complicated by hypertensive disorders of pregnancy (HDP).^1^ This includes 3–5% gestational hypertension (GH), characterised by new onset hypertension ≥20 weeks gestation, and 2–5% preeclampsia (PE), where new onset hypertension is associated with maternal organ dysfunction or foetal compromise.^1–5^ For affected mothers, GH and PE carry an increased lifetime risk of cardiometabolic diseases including hypertension, ischaemic heart disease, stroke, and type 2 diabetes.^3,5–10^

PE is also associated with adverse foetal outcomes such as foetal growth restriction (FGR), placental abruption, stillbirth and neonatal mortality. Approximately 12–33% of PE-exposed neonates are born small for gestational age (SGA).^11,12^ As delivery is the only definitive treatment for PE,^13^ many neonates are born preterm (<37 weeks gestation), with associated complications including respiratory distress syndrome and sepsis,^6,14–17^ and poorer outcomes are more likely for neonates exposed to early-onset PE (diagnosis <34 weeks gestation).^18,19^ While GH alone is associated with less severe perinatal outcomes,^20^ 25% of GH cases develop into PE, with a higher rate in early onset.^13,21^ In the long term, intrauterine exposure to HDP has been associated with an increased incidence of cardiometabolic,^22–25^ immunological^26–29^ and neurodevelopmental^30–37^ morbidities in children.

The Developmental Origins of Health and Disease (DOHaD) hypothesis provides a plausible explanation for the long-term complications seen in children exposed to HDP. This hypothesis suggests that foetal adaptations to an adverse intrauterine environment, as experienced in HDP, may cause ‘developmental programming’ that increases future risk of morbidity compared to children of normotensive pregnancies (NTPs).^38–40^ Rapid growth trajectories during infancy may also occur in response to an adverse intrauterine environment, typically nutrient insufficiency, a phenomenon known as the ‘thrifty phenotype’.^41^ Such rapid weight gain has been associated with later neurological, cardiovascular, renal and respiratory morbidity,^42,43^ as well as an increased risk of obesity and later cardiometabolic dysfunction.^44–46^

Our recent narrative review identified 11 studies assessing growth from birth to 2-years in infants exposed to PE versus NTP.^47^ Overall, studies reported that PE-exposed infants either had lower weight, length and BMI at 2 years than normotensive controls, or that they instead experienced accelerated weight gain to catch up in growth by 2 years.^47^ Combined with these inconsistent findings is the fact that the role of SGA and prematurity status within these studies was inconsistently explored. For instance, several prior studies were limited to HDP-exposed infants born premature or of very-low-birthweight (VLBW),^48–51^ which themselves are independent risk factors for impaired infant growth.^52,53^ Further research adjusting for these confounders is required and no prior study has investigated the difference in growth trajectories in a cohort comprising NTP, GH and PE-exposed infants in the first two years of life.

Our previous study^54^ reported lower absolute weight and weight z-scores in PE exposed compared with NTP infants at 6 months of age. The present study extends this research by comparing anthropometric growth measures and growth trajectories, including rapid weight gain and conditional weight gain, in this cohort at 2-years of age following exposure to NTP, GH or PE. Furthermore, we aim to elucidate impacts on anthropometric outcomes independent of SGA and prematurity status.

## Methods

### Study design

This is a sub-study of the prospective, single-centre cohort study, the P4 (Postpartum Physiology, Psychology and Paediatric) follow-up study being conducted at St. George Hospital, a metropolitan teaching hospital in Sydney, Australia. The study was approved by the Prince of Wales Hospital Human Research Ethics Committee (HREC/12/POWH395). A detailed study protocol has been published,^55^ and this study follows published papers reporting maternal outcomes^6^ and infant growth outcomes^54^ 6 months postpartum.

### Study population

Participants in this study were infants of P4-participating mothers. Mothers were eligible if they gave birth to a live singleton infant without major congenital abnormalities between January 2013 and December 2018 and had a good understanding of written and spoken English. Women were excluded from participating in the P4 study if they were pregnant again at the time of the 6 month postpartum assessment, or if they had pre-existing diabetes, hypertension, renal or any other serious maternal disease prior to the index pregnancy. Written informed consent for the mother and infant was obtained at study enrolment, which occurred by 6 months postpartum.

The P4 study initially recruited 415 women who were grouped based on their final diagnosis as either NTP (*n* = 302), GH (*n* = 23) or PE (*n* = 90) according to the International Society for the Study of Hypertension in Pregnancy (ISSHP) Guidelines.^21^ GH was defined as persistent, new onset hypertension (Blood Pressure (BP) ≥140 mmHg systolic or ≥90 mmHg diastolic) at or after 20 weeks gestation, while PE was new onset hypertension accompanied by evidence of maternal organ dysfunction including proteinuria, acute kidney injury, liver dysfunction, neurological features, haematological complications, and/or uteroplacental dysfunction.^21^

**Fig. 1:**
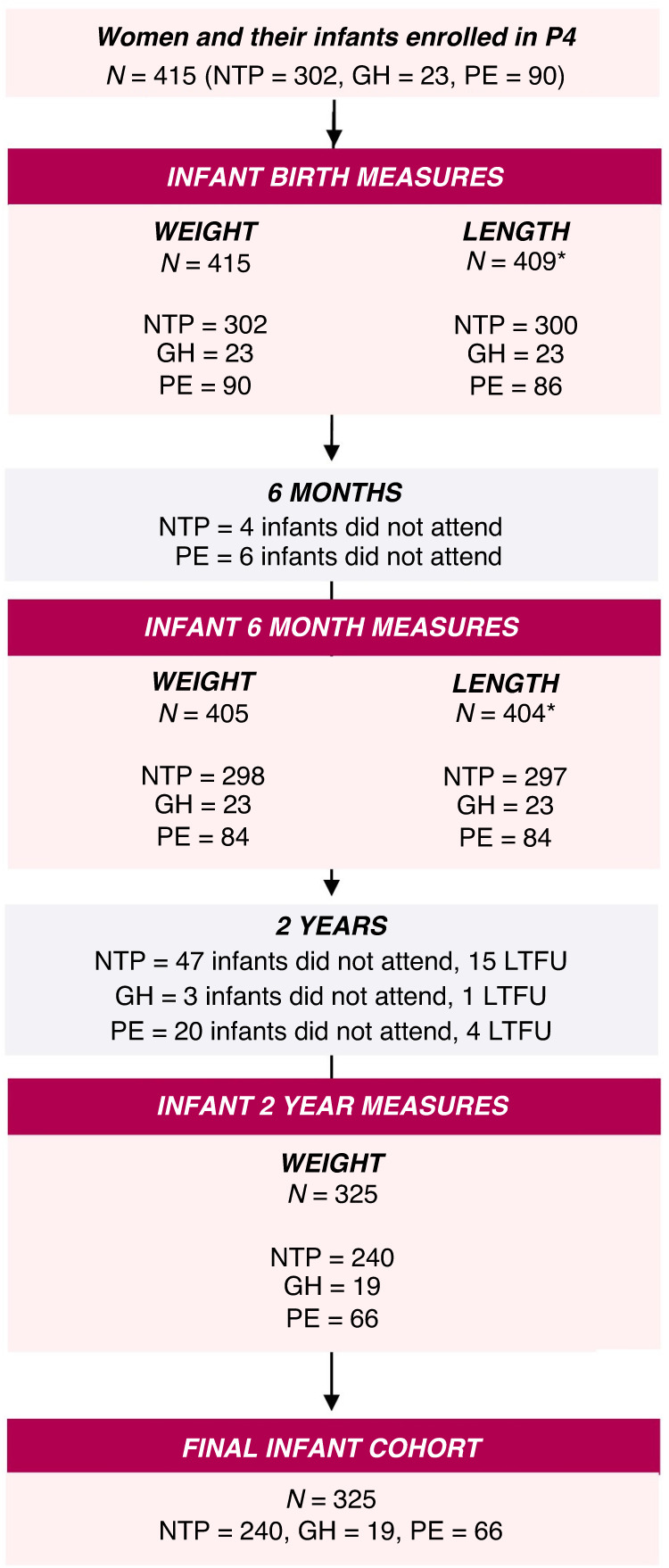
Flow diagram of infants participating in the P4 cohort study. GH gestational hypertension, LTFU loss to follow up, N number, NTP normotensive pregnancy, PE preeclampsia. *Differences in number of lengths recorded at birth and 6 months occurred due to incomplete or incorrectly recorded measurements.

This study includes infants exposed to NTP, GH or PE that had weight recorded at 2 years (Fig. 1). Prematurity status was defined as birth before 37 weeks gestation,^21^ and SGA status as a birthweight z-score corrected for sex and gestational age less than −1.28 (corresponding to below the 10^th^ percentile).^56,57^

### Data collection

Birthweight to the nearest gram and length to the nearest 0.5 cm were retrieved retrospectively from the mother’s electronic medical record. Weight, length and head circumference were measured at 6 months and weight and head circumference measured at 2 years, corrected for gestational age at birth, by a paediatrician.^58^ Weight in kilograms to the nearest one decimal place was measured with the infant lightly clothed without shoes using digital scales. Recumbent length at 6 months was measured with a measuring tape to the nearest 0.5 cm. Head circumference around the occipitofrontal diameter was measured with a measuring tape to the nearest millimetre.

Maternal sociodemographic information, medical history, birth details and all other details considered covariates, including total months breastfed, were collected both retrospectively from the mother’s electronic medical record, and prospectively from P4 surveys and study visits at 6 months and 2 years postpartum. Maternal BP, body composition, and metabolic markers were also collected at these study visits.

### Outcomes

As previously described (Gow et al. (2021)^54^), birth and 6-month weight and length z-scores were calculated for term infants using the World Health Organisation (WHO) Child Growth Standards,^59,60^ and for preterm infants using the INTERGROWTH-21st Preterm Postnatal Growth Standards,^61^ which allow for the differing pattern of postnatal growth to 6 months that preterm infants experience.^61^ The INTERGROWTH-21st standards converge with the WHO Child Growth Standards at 6 months, and thus the WHO standards were used for all infants from 6 months corrected age.^59,61^ Two-year weight z-scores were calculated for all infants using the WHO Child Growth Standards.^59,60^

Secondary measures of longitudinal infant growth calculated from birth to 2 years, and from 6 months to 2 years, included change in weight z-score, rapid weight gain, defined as an increase in weight z-score ≥0.67,^62^ and conditional weight gain, which considers the potential confounding of age, sex, birthweight, and regression to the mean on z-score change. Conditional weight gain is calculated using the standardised residuals from the linear regression of the 2-year versus 6-month, and 2-year versus birthweight z-scores with age and sex included as covariates. A positive value indicates a faster (and negative value a slower) rate of weight gain compared to the age and sex-adjusted population mean, adjusted for by the previous measurement to account for possible regression to the mean.^54,63,64^

### Covariates

Maternal, infant and birth data considered covariates in relation to GH or PE exposure and infant growth outcomes included:

- Maternal data: age, parity, smoking history, gestational diabetes mellitus status, maternal and paternal ethnicity and education, and first trimester, 6-month and 2-year weight, BMI, average systolic and diastolic BPs, Homoeostatic Model Assessment for Insulin Resistance (HOMA-IR) scores and Edinburgh Postnatal Depression Scale (EPDS) scores.
- Birth data: labour onset and mode of birth.
- Infant data: sex, birth gestation, prematurity status, SGA status, length of any neonatal intensive care unit or special care nursery (NICU/SCN) stay, and total months breastfed in the first 2 years.

### Statistical analysis

Descriptive statistics were used to summarise infant growth outcomes and covariates for the three exposure groups (NTP, GH or PE). One-way Analysis of Variance tests (parametric continuous data) and Kruskal-Wallis tests (non-parametric continuous data) with Tukey Post-Hoc analysis, and Chi-Square or Fisher’s Exact tests (categorical data) were used to estimate whether infant growth outcomes and covariates differed between groups. Associations between covariates and infant growth outcomes (i.e., weight, weight z-scores, z-score changes, rapid-weight-gain and conditional weight gain z-scores) were explored using simple linear regression. Significant associations were explored in multivariable linear regression, including adjustment for covariates and confounders such as SGA and prematurity status. Subgroup analyses stratified by SGA and prematurity status were conducted for selected growth outcomes significant in simple regression. Statistical analysis was performed using IBM SPSS Statistics, version 26.0 (Chicago, IL). A *p* value of <0.05 was considered statistically significant.

## Results

From the 415 infants enroled in the P4 Study, 325 had a recorded 2-year weight (Fig. 1) and are included in this analysis. Those without 2-year weights did not differ from those included in the final sample in terms of gestation at birth, birth weight z-score, 6-month weight z-score, maternal age and BMI at 6 months postpartum and duration of NICU/ SCN stay. Table 1 details the parental demographic and maternal health outcomes of those included in this study. Compared to the NTP group, both GH and PE mothers had significantly higher systolic and diastolic BPs, HOMA-IR scores and BMI at all time points (except first trimester BMI for the PE group), while GH mothers had significantly higher weight at all timepoints. Less than one quarter (*n* = 14) of PE women experienced early onset PE (≤33 weeks), 16 experienced late onset PE (>33 and <37 weeks), 33 experienced PE at term (≥37 weeks), and 3 experienced PE postpartum.

**Table 1:** Parental demographic and maternal health details of infants with exposure to NTP, GH or PE.

Parental demographic and maternal health details	NTP (*n* = 240)	GH (*n* = 19)	PE (*n* = 66)	*P* value
Mean ± SD				
Age, years	35 ± 4	35 ± 4	34 ± 5	0.19
Average systolic blood pressure, mmHg				
First trimester^a^	110 ± 11	124 ± 7	116 ± 11	<0.01
6 months	104 ± 8	117 ± 13	113 ± 10	<0.01
2 years^b^	103 ± 9	118 ± 10	112 ± 12	<0.01
Average diastolic blood pressure, mmHg				
First trimester^a^	68 ± 8	78 ± 7	71 ± 9	<0.01
6 months	66 ± 6	75 ± 9	73 ± 8	<0.01
2 years^b^	66 ± 7	74 ± 7	72 ± 8	<0.01
Median (IQR)				
Maternal weight, kg				
First trimester^c^	63 (14)	77 (31)	64 (16)	0.01
6 months	65 (18)	81 (33)	69 (20)	<0.01
2 years^b^	63 (17)	79 (43)	67 (22)	<0.01
Maternal BMI, (kg/m^2^)				
First trimester^c^	23 (6)	30 (8)	24 (7)	<0.01
6 months	24 (7)	30 (12)	27 (8)	<0.01
2 years^b^	23 (7)	29 (11)	27 (8)	<0.01
Maternal HOMA-IR				
6 months^d^	0.94 (0.81)	1.87 (2.58)	1.36 (1.53)	<0.01
2 years^e^	1.30 (0.80)	1.65 (3.00)	1.80 (1.73)	0.02
N (%)				
Maternal ethnicity^f^				0.27
Caucasian	136 (57)	15 (79)	34 (52)	
Asian	50 (21)	1 (5)	14 (21)	
European	34 (14)	3 (16)	9 (14)	
Other	19 (8)	0 (0)	9 (14)	
Paternal ethnicity^g^				0.98
Caucasian	136 (57)	12 (67)	39 (60)	
Asian	34 (14)	2 (11)	9 (14)	
European	37 (16)	2 (11)	12 (18)	
Other	30 (13)	2 (11)	6 (10)	
Maternal tertiary education completed^f^	219 (92)	16 (84)	63 (96)	0.21
Paternal tertiary education completed^h^	211 (89)	16 (84)	58 (91)	0.65
Maternal ever smoked	63 (26)	5 (26)	22 (33)	0.52
Gestational diabetes mellitus	27 (11)	5 (26)	10 (15)	0.12
EPDS Score screening for depression				
Pregnancy ^i^ (median [IQR] 14 [3]wks)	3 (1)	1 (5)	2 (3)	0.30
6 months	4 (2)	0 (0)	5 (8)	0.13
2 years^j^	10 (4)	0 (0)	2 (3)	0.30
EPDS Question 10- non-0 score, *n (%)*				
Pregnancy*^k^	0 (0)	0 (0)	0 (0)	1.00
6 months	4 (2)	0 (0)	0 (0)	0.67
2 years^*l*^	3 (1)	0 (0)	0 (0)	1.00

*BMI* body mass index, *BP* blood pressure, *EPDS* Edinburgh Postnatal Depression Score, *GH* gestational hypertension, *HOMA-IR* Homoeostatic Model Assessment for Insulin Resistance, *IQR* interquartile range, *kg* kilograms, *m* metres, *mmHg* millimetres of mercury, *N* number, *NTP* normotensive pregnancy, *PE* preeclampsia, *SD* standard deviation, *wks* weeks.

Missing data: ^a^*n* = 115, ^b^*n* = 140, ^c^*n* = 219, ^d^*n* = 3, ^e^*n* = 145, ^f^*n* = 1, ^g^*n* = 4, ^h^*n* = 5, ^i^*n* = 24, ^j^*n* = 8, ^k^*n* = 27, ^l^*n* = 6.

*Median (IQR) gestation= 14 (3) weeks.

Birth and infant outcomes are described in Table 2. Compared to the NTP group, GH and PE mothers were less likely to experience spontaneous labour, more likely to deliver via elective or emergency caesarean section, and PE mothers were more likely to have been nulliparous in the index pregnancy. Compared to both the NTP and GH groups, PE-exposed infants were more likely to be born preterm, SGA and be admitted to a NICU/ SCN. Median duration of all NICU/ SCN admissions was 3 days (interquartile range 18), and ranged in length from 1 to 77 days. Infants from both hypertensive groups were breastfed for a shorter duration than those from NTP.

**Table 2:** Birth and infant details following intrauterine exposure to NTP, GH or PE.

	NTP (*n* = 240)	GH (*n* = 19)	PE (*n* = 66)	*P* value	OR (95%CI)^b^
**Birth details**					
*N (%)*					
Labour onset				<0.01	(Non-spontaneous)
Spontaneous labour	144 (60)	1 (5)	6 (9)		PE: 15 (6.2–36.1)
Induction of labour	74 (31)	13 (68)	42 (64)		GH: 27 (3.5–205.6)
No labour	22 (9)	5 (26)	18 (27)		
Mode of birth				<0.01	(Non- normal vaginal)
Normal vaginal	157 (65)	7 (37)	22 (33)		PE: 3.7 (2.1–6.7)
Assisted vaginal	37 (15)	2 (11)	13 (20)		GH: 3.2 (1.2–8.5)
Elective caesarean section	19 (8)	4 (21)	6 (9)		
Emergency caesarean section	27 (11)	6 (32)	25 (38)		
**Infant details**					
*N (%)*					
Male sex	123 (51)	9 (47)	32 (49)	0.90	
Nulliparous pregnancy	120 (50)	11 (58)	48 (73)	<0.01	PE: 2.7 (1.5–4.8)
Premature birth (<37 weeks)	14 (6)	0 (0)	22 (33)	<0.01	PE: 8.1 (3.8–17)
34–36 + 6 weeks	12 (5)	0 (0)	12 (18)		
<34 weeks	2 (1)	0 (0)	10 (15)		
SGA birth	20 (8)	2 (11)	16 (24)	<0.01	PE: 3.5 (1.7–7.2)
NICU/SCN Admission	32 (13)	1 (5)	35 (53)	<0.01	PE: 7.3 (4–13.5)
Median (IQR)					
Birth gestation, weeks	39.6 (1.8)	39.1 (1.6)	37.6 (3.2)	<0.01	
Months breastfed^a^	12 (12)	8 (13)	8 (13)	0.03	

*GH* gestational hypertension, *CI* confidence interval, *IQR* interquartile range, *N* number, *NICU/SCN* neonatal intensive care unit/special care nursery, *NTP* normotensive pregnancy, *OR* unadjusted odds ratio, *PE* preeclampsia, *SGA* small for gestational age.

Missing data: ^a^*n* = 12.

^b^OR (95% CI) refer to the PE or GH group compared to the NTP group; Labour onset: NTP non-spontaneous labour is reference group; Mode of birth: NTP non-normal vaginal is reference group.

**Fig. 2:**
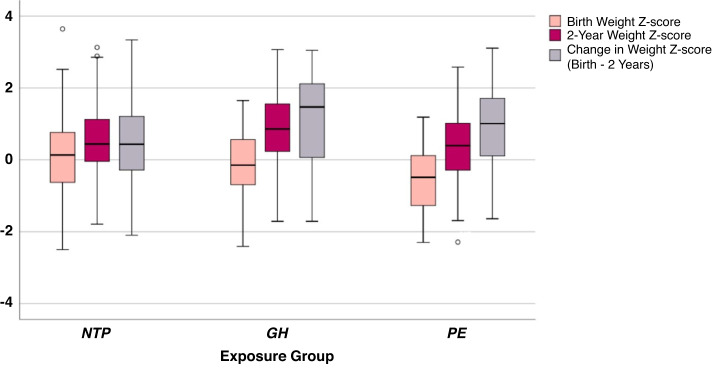
Birth and 2-year weight z-scores, and change in weight z-score from birth to 2 years versus exposure group. GH gestational hypertension, NTP normotensive pregnancy, PE preeclampsia. * Indicates a *p* value < 0.05 between groups. Error bars represent the 95% confidence interval.

Table 3 details infant growth outcomes after exposure to NTP, GH or PE. Compared to the NTP or GH groups, PE-exposed infants had lower weight, length and corresponding z-scores at birth (all *p* < 0.001). There were no significant differences in growth outcomes between groups at 6 months or 2 years. From 6 months to 2 years however, GH infants had significantly greater weight gain (*p* = 0.034), but there were no other significant differences between groups in this period. From birth to 2 years, GH and PE infants experienced significantly greater gains in weight and weight z-scores compared with NTP infants (Fig. 2), and were 2.4 [95% CI: 0.9–6.2] and 2.0 [1.1–3.5] times, respectively, more likely to experience rapid weight gain. In PE infants, weight gain and weight z-score gain from birth to 2 years in those exposed to early onset PE was 10.7 ± 1.3 kg and 0.52 ± 0.86, respectively. In PE infants exposed to late/ term/ postpartum weight gain and weight z-score change from birth to 2 years was 9.8 ± 1.5 kg and 1.04 ± 1.06, respectively. These differences were not statistically significant.

**Table 3:** Infant growth outcomes following intrauterine exposure to NTP, GH or PE.

Infant growth outcomes	NTP (*n* = 240)	GH (*n* = 19)	PE (*n* = 66)	*P* value
Median (IQR)				
Age at growth assessment, months				
6-month assessment^a^	6.4 (0.9)	6.2 (0.7)	6.8 (1.2)	<0.01
2-year assessment	24.5 (1.2)	24.5 (1.8)	24.8 (1.2)	0.10
Mean ± SD				
Weight, kg				
Birth	3.4 ± 0.5	3.4 ± 0.5	2.7 ± 0.7	<0.01
6 months^a^	7.9 ± 0.9	7.9 ± 1.1	7.7 ± 1.1	0.12
2 years	12.9 ± 1.5	13.5 ± 1.9	12.7 ± 1.7	0.12
Weight z-scores (corrected for gestational age ≤6 months)				
Birth	0.09 ± 0.98	−0.16 ± 1.03	−0.53 ± 0.85	<0.01
6 months^a^	0.07 ± 0.89	0.11 ± 1.09	−0.19 ± 0.99	0.10
2 years	0.54 ± 0.87	0.87 ± 1.13	0.40 ± 0.97	0.13
Length, cm				
Birth^b^	50.4 ± 2.7	51.1 ± 3.1	47.7 ± 4.2	<0.01
6 months^c^	68.4 ± 2.9	68.3 ± 2.4	67.7 ± 2.9	0.28
Length z-scores (corrected for gestational age ≤ 6 months)				
Birth^b^	0.10 ± 1.10	0.30 ± 1.55	−0.33 ± 0.94	0.01
6 months^c^	0.38 ± 1.25	0.55 ± 0.97	0.13 ± 1.06	0.26
Head circumference, cm				
6 months^c^	43.7 ± 1.5	43.5 ± 1.6	43.5 ± 1.6	0.47
2 years^b^	48.9 ± 1.5	49.0 ± 1.8	48.6 ± 1.5	0.25
Change in weight (6 months—2 years)				
Weight gain, kg^a^	5.0 ± 1.1	5.7 ± 1.3	5.0 ± 1.2	0.03
Change in weight z-score^a^	0.46 ± 0.75	0.76 ± 0.86	0.59 ± 0.80	0.16
Rapid weight gain, n (%)^a^	88 (37)	11 (58)	28 (42)	0.30
Conditional weight gain z-score^**a**^	−0.04 ± 0.97	0.44 ± 1.15	0.22 ± 1.02	0.13
Change in weight (birth—2 years)				
Weight gain, kg	9.5 ± 1.4	10.2 ± 1.9	10.0 ± 1.5	<0.01
Change in weight z-score	0.45 ± 1.07	1.03 ± 1.36	0.93 ± 1.10	<0.01
Rapid weight gain, *n (%)*	101 (42)	12 (63)	39 (59)	0.02
Conditional weight gain z-score	−0.05 ± 0.96	0.46 ± 1.25	0.04 ± 1.03	0.09

*BMI* body mass index, *cm* centimetres, *GH* gestational hypertension, *IQR* interquartile range, *kg* kilograms, *m* metres, *N* number, *NTP* normotensive pregnancy, *PE* preeclampsia, *SD* standard deviation.

Missing data: ^a^*n* = 3, ^b^*n* = 5, ^c^*n* = 4.

**Fig. 3:**
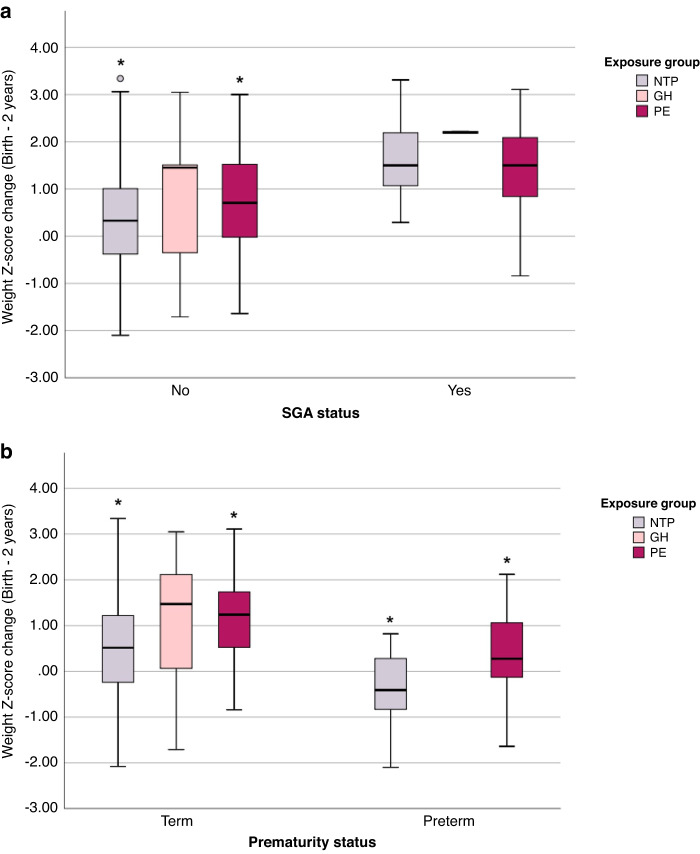
Change in weight z-score from birth to 2 years of infants with NTP, GH or PE exposure by SGA or prematurity status. **A** Change in weight z-score by SGA status. **B** Change in weight z-score by prematurity status. GH gestational hypertension, NTP normotensive pregnancy, PE preeclampsia, SGA small for gestational age. * Indicates a *p* value < 0.05 between groups. Error bars represent the 95% confidence interval.

When stratifying our cohort, infants born SGA experienced greater weight z-score gains than those born not SGA (*p* < 0.001). Similarly, infants born at term experienced greater weight z-score gains than those born preterm (*p* = 0.002). In infants not born SGA, PE-exposure remained significantly associated with higher weight z-score gain than NTP-exposed infants (*p* = 0.023) from birth to 2-years, while GH-exposed infants did not significantly differ from NTP-exposed infants. In both term and preterm infants, PE-exposed infants had higher weight z-score gains than NTP-exposed infants (term: *p* < 0.001, preterm: *p* = 0.014) (Table 4A, B, Fig. 3A, B).

**Tables 4:** A and B: NTP, GH and PE exposure and SGA status (4 A) or prematurity status (4B) versus change in weight z-score from birth to 2 years.

A						
	Non-SGA			SGA		
Exposure	N (%)	Mean ± SD	*P* value	N (%)	Mean ± SD	*P* value
NTP	220 (92)	0.337^a^ ± 1.01	<0.01	20 (8)	1.721 ± 0.85	0.46
GH	17 (89)	0.892 ± 1.37		2 (11)	2.200 ± 0.03	
PE	50 (76)	0.761^a^ ± 0.99		16 (24)	1.448 ± 1.02	
Any	287 (88)	0.441 ± 1.04	<0.01	38 (12)	1.631 ± 0.91	<0.01

B						
	Term			Preterm		
Exposure	N (%)	Mean ± SD	*P* value	N (%)	Mean ± SD	*P* value
NTP	226 (94)	0.503^b^ ± 1.06	<0.01	14 (6)	−0.417 ± 0.84	0.01
GH	19 (100)	1.030 ± 1.36		0 (0)	–	
PE	44 (66)	1.213^b^ ± 0.99		22 (33)	0.358 ± 0.90	
Any	289 (89)	0.646 ± 1.10	<0.01	36 (11)	0.057 ± 0.94	<0.01

*GH* gestational hypertension, *N* number, *NTP* normotensive pregnancy, *PE* preeclampsia, *SD* standard deviation, *SGA* small for gestational age.

^a^NTP and PE groups were significantly different (*p* = 0.023 in Post Hoc analysis).

^b^NTP and PE groups were significantly different (*p* < 0.001 in Post Hoc analysis).

In univariate linear regression, GH and PE exposure, SGA and prematurity status, 6-month maternal systolic and diastolic BPs, nulliparity, induction of labour and months breastfed to 2 years were all significantly associated with change in weight z-score from birth to 2 years (Supplementary Table 1). Six-month maternal diastolic BP and induction labour onset were highly associated with other variables significant in univariate linear regression and were subsequently excluded from multivariable regression.

Table 5 details multiple regression models that explore the influence of GH and PE exposure on change in weight z-score from birth to 2 years. When adjusting for SGA status (Model 1), both GH and PE exposure remained significantly associated with a greater increase in weight z-score compared to NTP exposure. After adjustments for total months breastfed (Model 2) and other maternal and birth covariates including 6-month maternal systolic BP, nulliparity status and prematurity status (Models 3) and all covariates (Model 4), only PE remained significantly associated with increases in weight z-score.

**Table 5:** Multivariable linear regression: unadjusted and adjusted models of the associations between GH and PE compared to NTP exposure versus change in weight z-score from birth to 2 years.

Expo-sure	Unadjusted		Model 1: adjusted for SGA status		Model 2: adjusted for total months breastfed to 2 years^a^		Model 3: adjusted for maternal and birth covariates^b^		Model 4: adjusted for all covariates^a,c^	
	Regression coefficient B (95% CI)	*P* value	Regression coefficient B (95% CI)	*P* value	Regression coefficient B (95% CI)	*P* value	Regression coefficient B (95% CI)	*P* value	Regression coefficient B (95% CI)	*P* value
GH	0.58 (0.08–1.09)	0.03	0.56 (0.08–1.03)	0.02	0.45(−0.06 to 0.96)	0.09	0.31 (−0.20 to 0.82)	0.24	0.18 (−0.31 to 0.67)	0.46
PE	0.48 (0.18–0.77)	<0.01	0.30 (0.02–0.59)	0.04	0.43 (0.13–0.72)	<0.01	0.55 (0.22 to 0.87)	<0.01	0.35 (0.03–0.66)	0.03

*CI* confidence interval, *GH* gestational hypertension, *N* number, *NTP* normotensive pregnancy, *PE* preeclampsia, *SGA* small for gestational age.

^a^*N* = 325 for Unadjusted, Models 1 and 3. *N* = 313 for Models 2 and 4.

^b^Model 3: adjusted for maternal covariates including 6-month systolic blood pressure and nulliparity status, and birth covariates including prematurity status.

^c^Model 4: adjusted for maternal covariates including 6-month systolic blood pressure and nulliparity status, birth covariates including SGA status and prematurity status, and postnatal covariates including total months breastfed to 2 years.

## Discussion

In our population of 325 infants, those exposed to PE were smaller at birth, but caught up to both NTP and GH-exposed infants by 6 months and 2 years. From birth to 2 years, both GH and PE-exposed infants experienced greater gains in absolute weight, weight z-score, and more rapid weight gain, but only PE-exposure remained significantly associated with this change in weight z-score after adjustment for confounding variables.

To our knowledge, we are the first to compare differences in infant growth and trajectories from birth to 2 years between NTP, GH and PE exposure groups. We found no association between GH or PE exposure and infant weight or weight z-scores at 6 months and 2 years compared with NTP. Our findings contrast with earlier studies of preterm and/or VLBW infants,^48,50,51^ and more recent studies in mixed cohorts,^54,65^ where those exposed to PE had lower absolute weight or weight z-scores throughout infancy. Martikainen et al.^49^ also reported this trend in preterm infants, however found no difference in weight in term infants of either exposure. This trend is more likely to reflect the impact of early-onset or severe PE, which are associated with greater uteroplacental dysfunction leading to preterm birth.^18,66^ Compared to previous studies, PE in our cohort was less severe and less preterm (only 10 (15%) of PE-exposed infants were born before 34 weeks), consistent with the expected rate in an unselected population. Furthermore, earlier studies did not adjust for preterm birth, SGA or VLBW status, common comorbidities experienced by PE-exposed infants, which are all independently associated with infant postnatal growth.^14,52,53^

Our findings support previous literature finding that PE-exposed infants ‘catch up’ to their normotensive counterparts in respect to growth outcomes.^67,68^ Correspondingly, we reported that PE-exposed infants experienced greater increases in weight z-score from birth to 2 years, independent of SGA and prematurity status. While FGR and subsequent SGA birth, common complications of PE, are associated with impaired infant growth,^14,52,53^ many SGA born neonates experience accelerated weight gain, possibly as a response to intrauterine undernutrition or clinical intervention where calories are added to feeds. This is referred to as ‘rapid catch-up growth’, an example of how infants born on weight extremes may experience natural regression to the mean postpartum.^44,63^ We reported this is our cohort from birth to 6 months,^54^ as well as in this study from birth to 2 years, irrespective of PE or NTP exposure. However, we also demonstrated that PE exposure is associated with accelerated infant weight gain, independent of confounders including prematurity and SGA status, which supports an intrauterine programming effect of PE as suggested by the DOHaD hypothesis. While adjusted confounders may reflect certain shared genetic predispositions and postnatal environmental factors such as infant feeding practices, we still cannot exclude residual confounding. Although conditional weight gain, which corrects for the regression to the mean that SGA infants may experience, approached significance from birth to 2 years, it was not different between groups, and thus further differentiation of the impact of SGA status is required in similar cohorts.

GH-exposed infants in our cohort did not differ significantly from NTP infants in weight, and while they experienced greater increases in weight z-score in infancy, this was not independent of perinatal confounders. This supports the hypothesis that shared lifestyle or genetic risk factors that predispose to both HDP and childhood morbidity, such as an adverse cardiometabolic profile, are responsible for their increased disease risk rather than an intrauterine programming effect.^8,22^ This may also explain why GH and late-onset PE, where there is less uteroplacental dysfunction, are associated with increased risks of childhood morbidity.^18,66^ Whether a result of GH and PE independently or in combination with these external factors, the increased growth trajectories and rapid weight gain experienced by exposed infants have nonetheless been associated with greater risks of obesity and cardiometabolic disease in later childhood.^46^ Furthermore, by age 20, Davis et al.^68^ described that those exposed to GH and PE causing preterm birth had a three-fold risk of hypertension, and those exposed to GH resulting in term birth had a two-fold risk of having obesity. Our findings suggest a need in primary care to identify accelerated growth trajectories in infants exposed to HDP and early screening for markers of cardiometabolic disease.^69^

Interventions to prevent accelerated weight gain trajectories could include strategies to increase the duration of breastfeeding, which is associated with reduced risk of future child and maternal cardiometabolic disease.^70^ In our study both hypertensive groups breastfed for a shorter duration compared to NTP. Furthermore, the introduction of greater nutrient-rich solid-feeding diets, strategies to combat fussy eating, and the encouragement of greater maternal and infant physical activity may improve future infant health outcomes.^54^

Strengths of this study include our ethnically diverse cohort, and similar to our previous study,^54^ our use of the INTERGROWTH-21^st^ Preterm Postnatal Growth Standards,^61^ which consider the differing postnatal growth patterns of preterm infants.^61^ Furthermore, our calculation of conditional weight gain was an additional outcome that considered the potential confounding of age, sex, birthweight, and regression to the mean on z-score change that SGA infants in our cohort may have experienced.^54,63,64^

Limitations of this study include the small sample size of GH infants that reduced statistical power and limited our ability to include further covariates in regression models, and the medium sample of PE infants that prevented sub-analyses based on PE onset or severity. Although SGA and prematurity status are common complications of PE, in some cases they are unrelated to PE, and adjustment for them in regression may have led to the overestimation of the impact of PE. We also lacked data on other confounding factors that may influence infant growth, including paternal factors such as height, weight, cardiometabolic profiles and other genetic or inheritable risk factors, postnatal infant solid feeding practices, activity levels, and the general postnatal environment including infections. Finally, the P4 Study was single-centred, and powered to detect differences in maternal BP outcomes 6 months after NTP, GH or PE exposure rather than paediatric outcomes specifically.

GH and PE are common obstetric complications with known long-term complications for mother and child. We demonstrated that HDP-exposed infants experience accelerated weight gain from birth to 2 years. For PE-exposed infants, this was independent of commonly comorbid intermediates including SGA and prematurity status, suggesting that PE may have an intrauterine programming effect. However, further research in larger, term-born, non-SGA cohorts is needed to further disentangle these covariates to understand the specific pathophysiological implications of GH and PE exposure. Nonetheless, these disorders are commonly comorbid to complications like SGA and prematurity status, and thus our findings provide opportunities for clinical intervention.

### Supplementary information

Supplementary Material

## Data Availability

The datasets generated and analysed during the current study are available from the corresponding author on reasonable request.
