# Supplementary material for: Childhood growth outcomes 2 years after hypertensive versus normotensive pregnancy: a P4 study
Source: Pediatr Res. 2023 Sep 6;95(1):275–84. doi: 10.1038/s41390-023-02789-7 (PMC10798880; doi:10.1038/s41390-023-02789-7)
Supplement: Supplementary file 1 — Supplementary Material [file 41390_2023_2789_MOESM1_ESM.pdf]

# Supplementary material

**Supplementary Table 1: Simple linear regression using maternal, birth and infant variables with significant differences between groups in the growth cohort to predict change in weight z-score from birth – 2 years.**

| *Variable* | $R^2$ | *Coefficient B* | *SEE* | *F-value* | *P-value* |
|---|---|---|---|---|---|
| SGA status | 0.12 | 1.19 | 1.03 | 44.86 | **<0.001** |
| Prematurity status | 0.03 | -0.59 | 1.08 | 9.47 | **0.002** |
| Hypertension exposure: | 0.04 | | 1.08 | 6.86 | |
| NTP (constant) | | 0.45 | | | |
| GH | | 0.58 | | | **0.025** |
| PE | | 0.48 | | | **0.002** |
| 6-month Maternal Weight* | 0.001 | 0.002 | 1.10 | 0.34 | 0.562 |
| 6-month Maternal BMI* | 0.001 | 0.004 | 1.10 | 0.12 | 0.732 |
| 6-month Maternal systolic blood pressure* | 0.04 | 0.02 | 1.08 | 12.30 | **0.001** |
| 6-month Maternal diastolic blood pressure* | 0.03 | 0.02 | 1.08 | 8.89 | **0.003** |
| 6-month Maternal HOMA-IR* | 0.001 | 0.02 | 1.10 | 0.41 | 0.523 |
| Nulliparity Status | 0.01 | 0.26 | 1.09 | 4.50 | **0.035** |
| Labour onset: | 0.06 | | 1.07 | 10.04 | **<0.001** |
| Spontaneous labour (constant) | | 0.33 | | | |
| Induction labour | | 0.57 | | | **<0.001** |
| No labour (caesarean section) | | 0.19 | | | 0.299 |
| Mode of Birth: | 0.005 | | 1.10 | 0.55 | 0.650 |
| Normal vaginal birth (constant) | | 0.54 | | | |
| Assisted vaginal | | 0.19 | | | 0.280 |
| Elective caesarean section | | 0.15 | | | 0.496 |
| Emergency caesarean section | | -0.02 | | | 0.894 |
| Birth gestation | 0.006 | -0.04 | 1.10 | 2.00 | 0.158 |
| NICU/SCN admission length | 0.01 | -0.01 | 1.09 | 3.98 | **0.047** |
| Months breastfed to 2 years | 0.07 | -0.04 | 1.07 | 21.65 | **<0.001** |

Abbreviations: BMI, body mass index; GH, gestational hypertension; HOMA-IR, Homeostatic Model Assessment for Insulin Resistance; NICU/SCN, neonatal intensive care unit/special care nursery; NTP, normotensive pregnancy; PE, preeclampsia; SEE, standard error of the estimate; SGA, small for gestational age.
*6-month maternal body composition and cardiometabolic outcomes were chosen for simple linear regression instead of trimester 1 outcomes as the 6-month outcomes were all collected by our study research midwife, whilst most of the trimester 1 outcomes were collected retrospectively from medical records of booking visits with the mother's general practitioner or healthcare provider. The 2-year outcomes were not chosen as Brown et al. [6] have previously concluded that 6-month and 2-year maternal outcomes followed a similar trend of being significantly higher among the PE versus NTP group.
